# Ionizing Radiation Induces Stemness in Cancer Cells

**DOI:** 10.1371/journal.pone.0043628

**Published:** 2012-08-21

**Authors:** Laura Ghisolfi, Andrew C. Keates, Xingwang Hu, Dong-ki Lee, Chiang J. Li

**Affiliations:** 1 Skip Ackerman Center for Molecular Therapeutics, Division of Gastroenterology, Beth Israel Deaconess Medical Center, Harvard Medical School, Boston, Massachusetts, United States of America; 2 Global Research Laboratory for RNAi Medicine, Department of Chemistry, Sungkyunkwan University, Suwon, Korea; Kanazawa University, Japan

## Abstract

The cancer stem cell (CSC) model posits the presence of a small number of CSCs in the heterogeneous cancer cell population that are ultimately responsible for tumor initiation, as well as cancer recurrence and metastasis. CSCs have been isolated from a variety of human cancers and are able to generate a hierarchical and heterogeneous cancer cell population. CSCs are also resistant to conventional chemo- and radio-therapies. Here we report that ionizing radiation can induce stem cell-like properties in heterogeneous cancer cells. Exposure of non-stem cancer cells to ionizing radiation enhanced spherogenesis, and this was accompanied by upregulation of the pluripotency genes Sox2 and Oct3/4. Knockdown of Sox2 or Oct3/4 inhibited radiation–induced spherogenesis and increased cellular sensitivity to radiation. These data demonstrate that ionizing radiation can activate stemness pathways in heterogeneous cancer cells, resulting in the enrichment of a CSC subpopulation with higher resistance to radiotherapy.

## Introduction

Cancer stem cells (CSCs), a subpopulation of malignant cells in the heterogeneous cancer cell population, are considered to be responsible for cancer recurrence, metastasis and drug resistance. CSCs have been isolated from a variety of human malignancies including leukemia [1], [2], breast cancer [3], [4], brain tumor [5], hepatocellular carcinoma [6], pancreatic cancer [7] and colorectal cancer [8], [9]. CSCs have the ability to self-renew and to differentiate into the multitude of cells that comprise the bulk of the tumor mass [10], [11]. CSCs also express high levels of drug resistance transporter proteins (e.g. ABC) [12], [13], [14], DNA repair enzymes [15], [16] and anti-apoptotic proteins [17], [18], [19], which renders them highly resistant to conventional cancer therapies including chemotherapy and radiation. For example, studies published by Bao et al [20] have demonstrated that ionizing radiation can enrich CD133+ glioma cancer stem cells *in vitro* and *in vivo*. Moreover, these authors showed that this enrichment effect was mediated by preferential activation of the DNA damage checkpoint in CD133+ glioma cancer stem cells compared to CD133- non-stem glioma cells. The CSC model, therefore, calls for the design of therapeutics that target CSCs to improve cancer treatment [21], [22].

Although there is increasing evidence to support the CSC hypothesis, the exact origin of these cells remains controversial. One possibility is that CSCs result from oncogenic transformation of normal tissue stem cells [23]. In this scenario, mutations in the regulatory mechanisms controlling stem cell self-renewal are thought to promote the formation CSCs [24], [25], which then generate a hierarchical and heterogeneous cancer cells, suggesting that the originating cancer cell has the capacity to generate multiple cell types (i.e. multidifferentiative plasticity), a hallmark of stem-like cells [26], [27], [28]. Alternatively, CSCs may be derived from non-stem cancer cells that have acquired stemness properties [22], [29]. In keeping with this, studies published by Quintana et al, and Roesch et al [30], [31] have shown that a CSC phenotype can be acquired by tumor cells previously negative for specific CSC markers.

In this study, our data suggest irradiation of cancer cells as a novel potential origin of cancer stemness. Exposure of heterogeneous cancer cells to ionizing gamma radiation enhanced spherogenesis under stem cell culture conditions. Surprisingly, irradiation of CSC-depleted heterogeneous cancer cell populations induced the emergence of sphere-forming cells. At the molecular level, analysis of the pluripotency gene expression following gamma irradiation showed up-regulation of Sox2 and Oct3/4 mRNA and protein. In contrast, knockdown of Sox2 or Oct3/4 markedly reduced surviving colonies following radiation treatment, and also significantly inhibited radiation–induced spherogenesis. These data demonstrate that radiation can activate stemness pathways in heterogeneous cancer cells, suggesting a novel mechanism of resistance of cancer cells to radiotherapy. They also imply that targeting of CSCs may improve the efficacy of radiotherapy.

## Results

### Gamma Radiation Increases Spherogenesis by Cancer Cells

**Figure 1:**
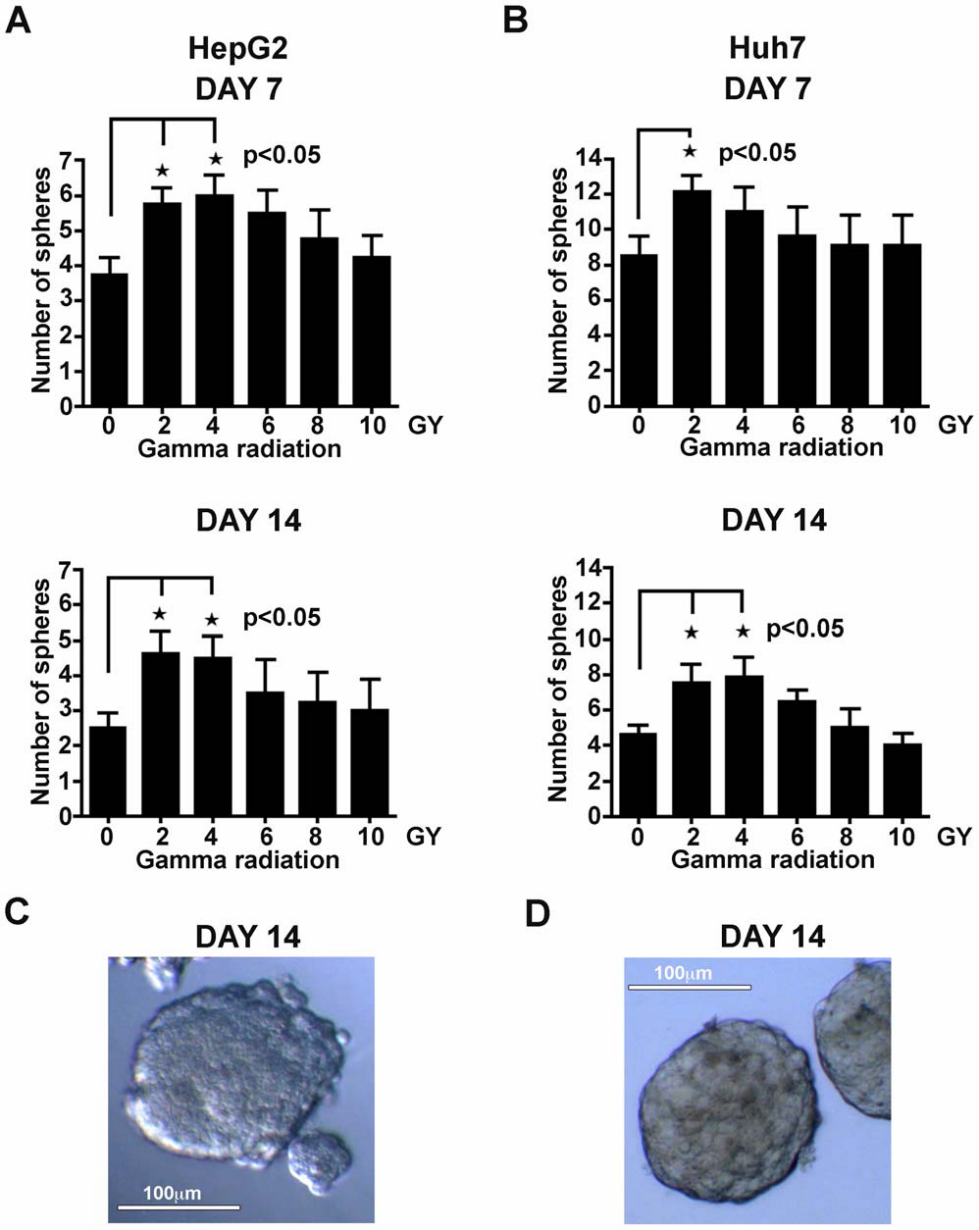
Ionizing radiation increases spherogenesis in HCC cells. **A and B** HepG2 cells (a) and Huh7 cells (b), were exposed to increasing doses of gamma radiation, and then seeded in stem cell media onto 96-well ultra low attachment plates at 500 cells/well. The sphere numbers were counted after 7 days (top) and 14 days (bottom) of culture, and relative numbers were reported on the graphs. Lower radiation doses of 2 and 4 Gy induced a significant increase in sphere formation in both cell lines compared to untreated samples. Results are presented as mean±SEM of four independent experiments. *p<0.05, **p<0.01 versus untreated control cells. **C and D** Representative images of HepG2 (c) and Huh7 (d) spheres formed after 14 days of culture in stem cell media. The images were captured using a digital camera (AmScope, iScope Corp., Chino, CA), mounted on a Zeiss Axiovert 25 inverted microscope. Magnification: 100x.

We first examined the effect of ionizing radiation on the ability of hepatocellular carcinoma cells, for which a CSC component has been previously described [6], [32], to grow as spheres under stem cell media (SCM) culture conditions. Single cell suspensions of HepG2 cells and Huh7 cells were exposed to 0–10 Gy of gamma radiation (for LD_50_ see Figure S1) and then seeded at clonal density onto ultra low attachment plates in serum-free SCM. Sphere formation was evaluated after 7 days and 14 days of culture. Both cell lines were able to form spheres (Figures 1c, 1d). As shown in Figures 1a and 1b, a 40–50% increase in the number of spheres was observed for HepG2 cells on day 7 and day 14, and for Huh7 cells on day 14 after treatment with 2 Gy or 4 Gy of gamma radiation. These findings show that ionizing gamma radiation can significantly increase the *in vitro* spherogenesis of HepG2 and Huh7 cells.

### Gamma Radiation Induces Spherogenesis in HepG2 and Huh7 Non-side Population Cells

Side population flow cytometry (defined by the ability to exclude the DNA-binding dye Hoechst 33342) [33], [34] has been used to enrich CSC and non-CSC from various cancer cell lines, as well as cultures derived from primary tumors [26], [35], [36]. This approach has shown that HepG2 and Huh7 CSCs represent ∼1–2% of the bulk tumor cells [6], [32]. Given that the ability to form spheres *in vitro* under non-adherent culture conditions is considered a property of CSCs [32], [37] our data strongly indicate that gamma irradiation of HepG2 and Huh7 cells significantly increased in the number of CSCs in both cell lines.

**Figure 2:**
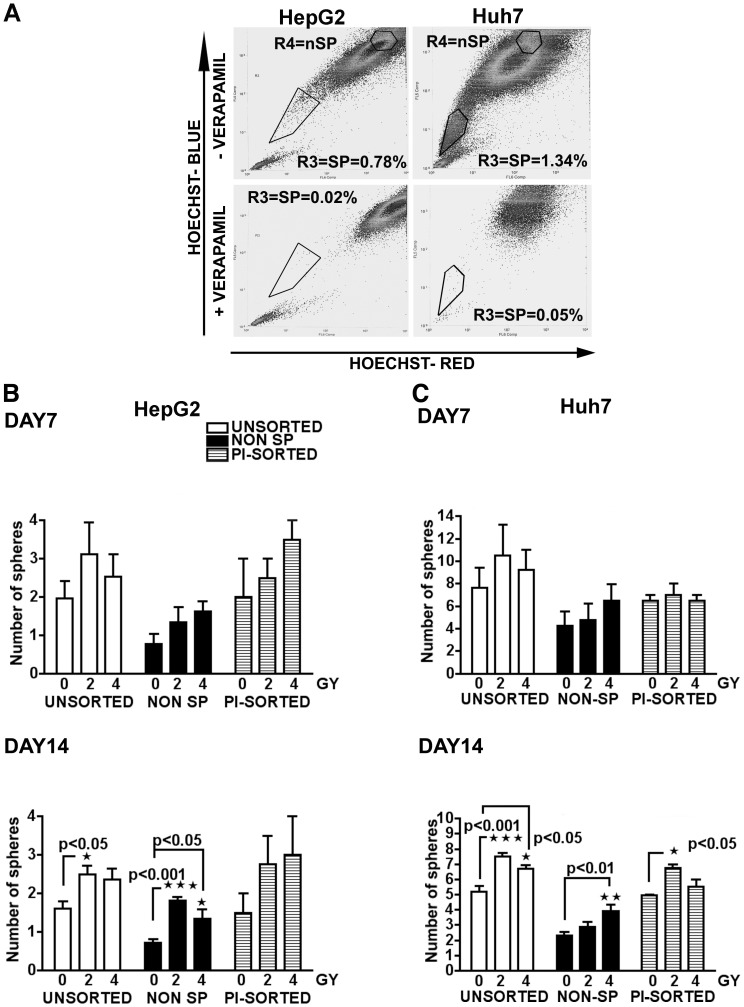
Ionizing radiation increases spherogenesis in the non-Side Population fraction of HCC cells. **A** HepG2 cells (left panels) and Huh7 cells (right panels) were stained using Hoechst 33342 with (lower panels) or without (upper panels) Verapamil, and then sorted using a MoFLO2 fluorescence activated cell sorter. The R3 gate identified the Side Population (SP) fraction. Non-Side Population (Non-SP) cells, isolated via the R4 gate, were collected, rinsed in PBS and resuspended in stem cell media. **B and C** Unsorted, non-SP and PI-sorted HepG2 (b) and Huh7 (c) cells were exposed to 0, 2 or 4 Gy of gamma radiation and then seeded onto 96-well ultra low attachment plates at 500 cells/well. Sphere numbers were then counted after 7 days (top panels) and 14 days (bottom panels) of culture, and relative numbers were reported on the graphs. White bars represent unsorted tumor cells, black bars represent sorted non-side population (non-SP) cells, and hatched bars represent PI-sorted cells. After 14 days of culture in SCM, radiation doses of 2 and 4 Gy induced a significant increase in sphere formation in the bulk tumor population and in the non-SP population of both cell lines compared to untreated samples, while PI-sorted Huh7 cells showed significantly increased sphere formation following 2 Gy of radiation treatment. Results are presented as mean±SEM of five independent experiments (unsorted and non-SP populations) or mean±SEM of two independent experiments (PI-sorted population). *p<0.05, **p<0.01 versus untreated control cells.

To investigate whether the increased spherogenesis observed following exposure to gamma radiation might originate within the heterogeneous non-stem cancer cell population, we used side population flow cytometry to identify and isolate non-CSC from HepG2 and Huh7 cells. A typical non-side population sorting experiment is illustrated in Figure 2a. Cells sensitive to the efflux pump inhibitor verapamil (R3 gate) show low Hoechst staining intensity and were identified as the side population (SP) component of the tumor (i.e. CSC-enriched). Verapamil-insensitive cells with high Hoechst staining intensity (R4 gate) were isolated as non-side population (non-SP) cells. To exclude the possibility of non-specific effects on sphere formation resulting from the FACS sorting procedure, HepG2 and Huh7 cells were also mock-sorted based on propidium iodide (PI) staining. Following cell sorting in SCM, non-SP (i.e. CSC depleted) cells and control cells (unsorted bulk or PI-sorted HepG2 and Huh7) were irradiated with 0, 2 or 4 Gy of gamma radiation in SCM. Irradiated bulk, irradiated non-SP cells and irradiated PI-sorted cells were then seeded onto ultra low attachment plates and sphere formation was evaluated after 7 and 14 days of culture.

As shown in Figure 2b and Figure 2c, no significant difference in sphere formation was observed after 7 days of culture in SCM for unsorted, non-SP, or PI-sorted HepG2 or Huh7 cells subjected to 2 or 4 Gy of gamma radiation. In contrast, unsorted HepG2 cells exposed to 2Gy of radiation and unsorted Huh7 cells exposed to 2 or 4 Gy of radiation had significantly increased sphere formation after 14 days of culture in SCM (Figure 2b, 2c). Moreover, PI-sorted control cells from both cell lines showed similar sphere-forming ability to unsorted bulk HepG2 or Huh7 cells after 14 days of culture in SCM. Specifically, PI-sorted Huh7 cells subjected to 2 Gy of gamma radiation showed significantly increased sphere formation after 14 days of culture in SCM (p<0.05). PI-sorted HepG2 cells exposed to 2 or 4 Gy of radiation also displayed markedly elevated sphere formation after 14 days of culture in SCM. These differences, however, did not reach statistical significance. Surprisingly, exposure to gamma radiation markedly induced spherogenesis in the non-SP fractions from HepG2 and Huh7 cells after 14 days of culture in SCM. For HepG2 cells, treatment of the non-SP fraction with 2 Gy of gamma radiation induced a 150% increase in sphere formation compared to untreated non-SP cells (p<0.001). Similarly, treatment of Huh7 non-SP cells with 4 Gy of gamma radiation induced an 80% increase in sphere formation (p<0.01). Taken together, these data demonstrate that low dose gamma radiation can promote the formation of CSCs within the heterogeneous non-stem cancer cell population.

### Stemness Gene Expression is Increased in HepG2 and Huh7 Cells Following Gamma Radiation Treatment

**Figure 3:**
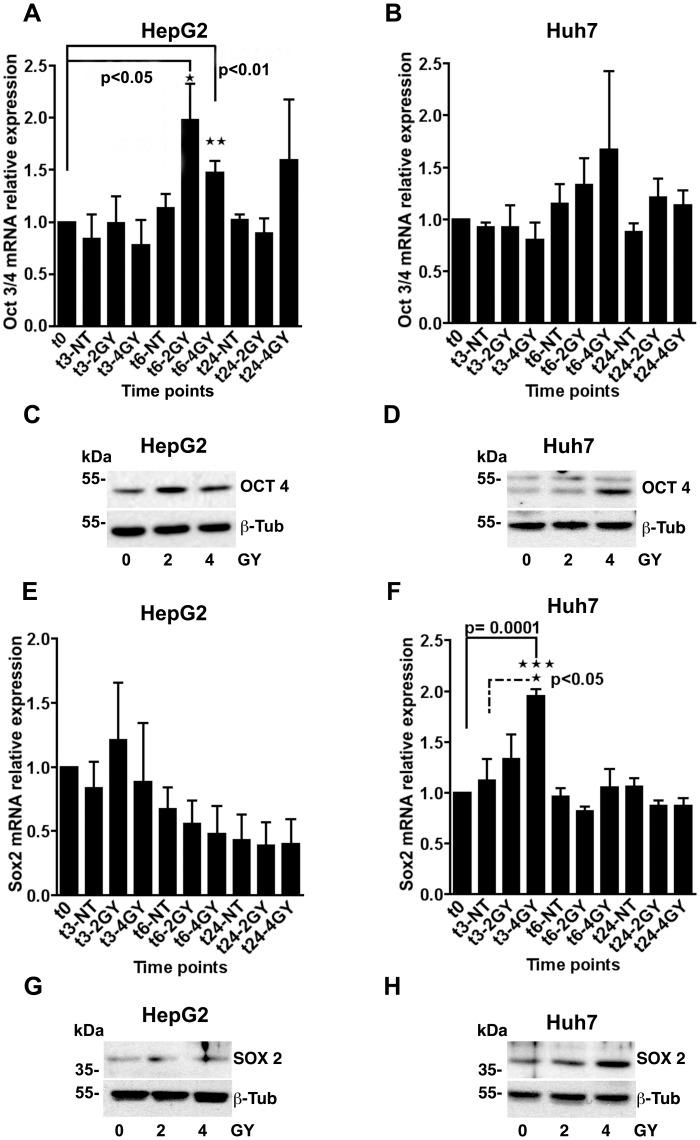
Upregulation of stemness genes in HCC cells after radiation treatment. **A and B, E and F** HepG2 and Huh7 cells were exposed to 0, 2 or 4 Gy of gamma radiation and total RNA was extracted after 3, 6 or 24 hours. Oct3/4 (a and b) and Sox2 (e and f) mRNA levels in each cell line were then determined by quantitative Real-Time PCR and normalized to GAPDH mRNA levels in each sample. In HepG2 cells, treatment with 2 and 4 Gy of gamma radiation induced a significant increase of Oct3/4 mRNA levels. Sox2 mRNA levels were also strongly upregulated in Huh7 cells following low dose gamma radiation treatment. Results are presented as mean±SEM of four independent experiments. *p<0.05, **p<0.01, ***p<0001 versus t0 sample or untreated samples. The dashed line represents a comparison to the control at the same time point. **C and D,** **G and H** HepG2 cells and Huh7 cells**,** were exposed to 0, 2 or 4 Gy of gamma radiation and Oct4 or Sox2 protein expression was determined by Western blot analysis after 6 hours (for Oct4; c and d), or after 4 hours (for Sox2; g and h). Oct4 and Sox2 protein levels increased following radiation treatment consistent with the increases in mRNA levels for each gene.

To examine if the increased spherogenesis induced by gamma radiation may be due to elevated stemness gene expression, HepG2 cells and Huh7 cells were exposed various doses of gamma radiation, and the level of Oct3/4 and Sox2 mRNA was evaluated by real-time PCR. As shown in Figure 3a and 3c, a significant increase in Oct3/4 mRNA and protein was detected in HepG2 cells 6 hours after exposure to 2 or 4 Gy of gamma radiation. Increased Oct4 protein levels were also observed in Huh7 cells 6 hours after exposure to 4 Gy of radiation (Figure 3d). Radiation–induced increases in the level of Huh7 cell Oct3/4 mRNA, however, did not reach statistical significance (Figure 3b).

Consistent with our findings regarding Oct3/4 expression, we found that Sox2 mRNA and protein levels were also significantly increased in Huh7 cells 3 and 6 hours after exposure to 4 Gy of radiation (Figure 3f, 3h). However, no increase in Sox2 mRNA and protein levels was detected in HepG2 cells following radiation treatment (Figure 3e, 3g). These results suggest that gamma radiation can induce the reprogramming of differentiated cancer cells to a more stem-like phenotype by inducing stemness gene expression.

### Oct3/4 and Sox2 Knockdown Sensitizes HepG2 and Huh7 Hepatocellular Cancer Cells to Gamma Radiation

**Figure 4:**
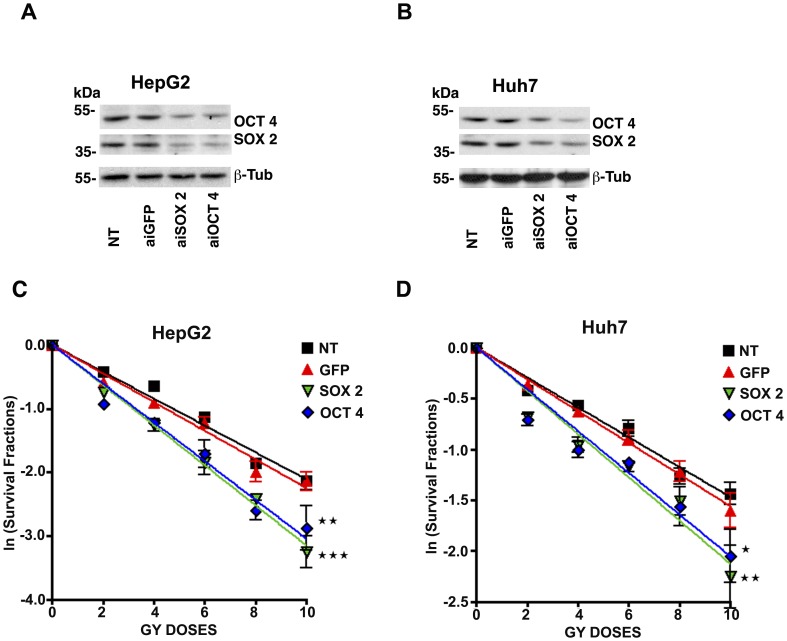
Silencing of stemness genes increases the sensitivity of hepatocellular carcinoma cells to gamma radiation. **A and B** HepG2 cells (a) and Huh7 cells (b), were transfected with 100 nM of aiRNA targeting GFP, Sox2 or Oct4, and knockdown efficiency was evaluated by Western blot after 48 hours. Sox2 and Oct4 belong to the same regulatory circuit; therefore, single gene knockdown leads to reduced expression levels of both proteins. **C and D** HepG2 and Huh7 cells transfected with aiRNA targeting GFP, Sox2 or Oct4 were irradiated with 0, 2, 4, 6, 8 or 10 Gy of gamma radiation and equal numbers of cells were plated onto 6-well plates for colony formation assay. On day 7, colonies were counted and the fraction of surviving clonogenic cells expressed as a natural log was plotted. Lines are fitted using a first-order polynomial regression and represent the mean of four independent experiments. *p<0.05, **p<0.01, ***p<0.001 versus GFP-transfected cells.

Since Sox2 and Oct3/4 upregulation correlates with increased stemness (spherogenesis) in HepG2 and Huh7 cells following exposure to gamma radiation, we next examined whether these factors could affect the ability of HepG2 or Huh7 cells to resist radiation treatment. For these experiments, Sox2 or Oct4 gene expression was silenced in Huh7 and HepG2 cells using asymmetric interfering RNA (aiRNA). aiRNA were chosen, instead of siRNA, due to their superior specificity [38]. Knockdown efficiency was evaluated by Western blot 48 h after aiRNA transfection. aiRNA targeting Sox2 or Oct4 efficiently reduced the expression of both proteins (Figure 4a and 4b). This is consistent with previous studies using embryonic stem cells that demonstrate Oct4 and Sox2 are linked to the same regulatory pathway, which includes auto-regulatory loops and reciprocal auto-transcription regulation [39], [40]. Single gene knockdown within the Sox2-Oct4 regulatory circuit would, therefore, be expected to reduce the expression level of both proteins.

To assess the effect of Sox2 or Oct4 knockdown on cell viability following radiation treatment, Huh7 and HepG2 cells were transfected with aiRNA targeting Sox2, Oct4 or GFP. After 24 h, the cells were divided and exposed to 0, 2, 4, 6, 8 or 10 Gy of gamma radiation. Single cell suspensions were then seeded in complete DMEM in standard 6-well plates to allow the cells to attach and form colonies. After 7 days of culture in complete DMEM, the colonies formed under each treatment were stained and counted. As shown in Figure 4c and 4d, silencing of Sox2 or Oct4 gene expression in HepG2 and Huh7 cells resulted in a significant increase in sensitivity to gamma radiation (decreased LD_50_ value; see Table 1) when compared to cells transfected with an aiRNA directed against GFP, or non-transfected cells. These data suggest that downregulation of stemness genes can sensitize hepatocellular carcinoma cells to gamma radiation treatment.

**Table 1:** Mean slope ± SEM and LD_50_ value for each treatment.

CELL LINE	SAMPLE	SLOPE ± SEM	LD_50_
HepG2	NT	−0.21±0.0063	3.24
	GFP	−0.22±0.0072	3.08
	SOX 2	−0.31±0.0081	2.29
	OCT 4	−0.30±0.0115	2.33
Huh7	NT	−0.15±0.0048	4.68
	GFP	−0.16±0.0053	4.41
	SOX 2	−0.21±0.0101	3.27
	OCT 4	−0.20±0.0098	3.36

Slope values are expressed as the natural log of the colony survival fraction. The mean of four independent experiments is shown.

### Oct3/4 and Sox2 Knockdown in HepG2 or Huh7 Cells Inhibits Radiation–induced Sphere Formation

**Figure 5:**
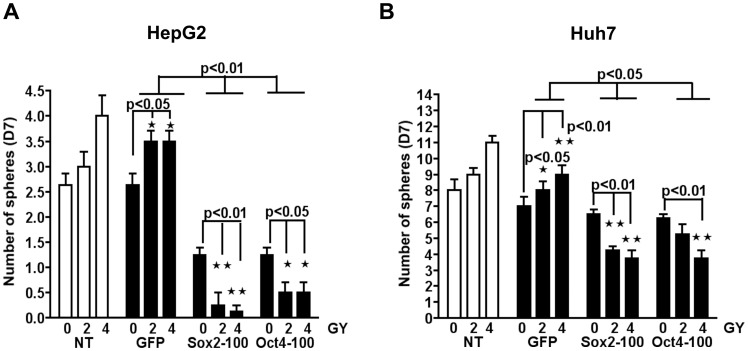
Downregulation of Sox2 and Oct3/4 expression inhibits sphere formation induced by radiation treatment. **A and B** HepG2 cells and Huh7 cells transfected with aiRNA targeting GFP, Sox2 or Oct4 were exposed after 24 hours to 0, 2 or 4 Gy of gamma radiation and then plated onto 96-well ultra low attachment plates at 500 cells/well in stem cell media. Sphere numbers for each knockdown group and radiation treatment were recorded on day 7 of culture in stem cell media. Silencing of Sox2 or Oct4 significantly reduced sphere formation in HepG2 and Huh7 cells treated with low doses of gamma radiation compared to non-irradiated cells, or cells transfected with aiRNA against GFP. Results are presented as mean±SEM of four independent experiments, n = 4. *p<0.05, **p<0.01, versus GFP-transfected cells or non-irradiated control cells (0 Gy).

Since knockdown of Sox2 or Oct4 increased the sensitivity of HepG2 and Huh7 cells to radiation treatment, we next examined whether the spherogenesis ability of HepG2 and Huh7 cells following gamma irradiation was associated with the expression of these factors. To assess the effect of radiation treatment, Huh7 and HepG2 cells were transfected with aiRNA directed against Sox2, Oct4 or GFP, harvested after 24 h, and then divided and exposed to 0, 2 or 4 Gy of gamma radiation. Cells were then seeded at clonal density onto ultra-low attachment plates in serum-free SCM. After 7 days of culture, gamma-irradiated cells transfected with aiRNA directed against GFP showed a similar increase in sphere formation to the untreated group. Huh7 and HepG2 cells treated with Sox2 or Oct4 aiRNA and exposed to radiation, however, formed significantly less spheres than control cells transfected with GFP aiRNA (Figure 5a and 5b). More importantly, cells treated with Sox2 or Oct4 aiRNA had a significantly reduced ability to form spheres following exposure to 2 or 4 Gy of gamma radiation, compared to non irradiated cells. In HepG2 cells, a significant inhibition of sphere formation was also observed in non-irradiated cells upon silencing of Sox2 or Oct4. Taken together, these results demonstrate that expression of Sox2 and Oct3/4 is required for CSC in HepG2 and Huh7 cells, and that upregulation of these factors in non-CSCs may be sufficient to induce the acquisition of a CSC phenotype, thereby imparting a higher radiation resistance to the bulk tumor cell population.

## Discussion

Cancer stem cells (CSCs) are thought to represent a small sub-population of cells present in most tumors that, similar to normal tissue stem cells, possess the ability to self-renew, to divide asymmetrically and symmetrically, and to undergo multi-lineage differentiation [41], [42]. These features of CSCs are fundamentally responsible for their unique ability to initiate and sustain tumors [10], [42], [43]. Moreover, CSCs are also believed to play a key role in cancer metastasis, cancer recurrence, and cancer drug resistance [15], [20], [44], [45].

In this study, we demonstrate that cancer cells can be induced, by gamma radiation, to acquire a stemness state characterized by increased stemness gene expression and a cancer stem cell-like phenotype. Side population flow cytometry has shown that CSCs in HepG2 and Huh7 represent approximately 1–2% of the bulk tumor cells [6], [32]. Given that the ability to form spheres in vitro under non-adherent culture conditions is specific to CSCs [32], [37], our data suggest that gamma irradiation of HepG2 and Huh7 cells significantly increased in the number of CSCs in both cell lines.

Recent publications have shown that, unlike bulk tumor cells, CSCs possess intrinsic resistance to radiation therapy *in vitro* and *in vivo* [20], [45], [46], and that this property most likely results from higher expression of free radical-removing enzymes, increased efficiency in DNA-damage repair, and preferential DNA-damage checkpoint activation [15], [16], [20], [47]. To further explore the origin of the increased numbers of CSCs in gamma irradiated HepG2 and Huh7 cells we performed flow cytometry using Hoechst 33342 dye exclusion to isolate side population (SP) cells that are enriched in CSC, and non-side population (non-SP) cells that are depleted of CSC. Surprisingly, we observed significantly increased sphere formation in HepG2 and Huh7 non-SP cells following exposure to 2 or 4 grey of gamma radiation (Figure 2). Moreover, increased sphere formation was also seen in untreated HepG2 and Huh7 non-SP cells. These findings indicate that non-SP cells (i.e. non-CSC tumor cells) can acquire CSC-like properties, and are consistent with the recent concept that tumors are comprised of a variety of cells at different maturation stages [48], [49], with the ability to convert into a more stem cell-like state [50], [51].

Previous studies have reported that radiation-induced enrichment of CSCs is associated with activation of self-renewal signaling pathways such as Wnt/β-catenin, Notch and Hedgehog [52], [53], [54]. Moreover, since CSCs are capable of both asymmetric and symmetric cell division [55], [56] the enrichment effect is thought to be mediated primarily by CSCs undergoing symmetric cell division. Our data, however, suggest that an additional component of this effect may be the acquisition of stemness characteristics upon radiation treatment by non-stem cancer cells. This finding is further supported by our observation of increased Sox2 and Oct3/4 pluripotency gene expression in hepatocellular carcinoma cells following gamma irradiation (Figure 3).

Along with c-Myc, Klf4 and NANOG, Sox2 and Oct3/4 transcription factors are considered key genes for the production of murine and human induced pluripotent stem cells [57], [58]. Specifically, Sox2 and Oct3/4 expression seems to have a fundamental role in ensuring the maintenance of self-renewal, plasticity and the reprogramming ability in both embryonic stem cells and CSCs [59], [60], [61], [62]. Increased expression of Sox2 and Oct3/4 following gamma radiation treatment (Figure 3) is, therefore, consistent with induction of a genetic program in some HepG2 and Huh7 cells that results in increased stemness, and the acquisition of a stem cell-like phenotype [63], [64], [65]. Since the CSC component in both cell lines represents ≤2% of the total cell population (Figure 2) [6], [32], the observed overexpression of Sox2 and Oct3/4 in HepG2 and Huh7 cells following low dose irradiation most likely represents changes in the non-CSC population.

In this study, we found that down regulation of Sox2 and Oct3/4 in HepG2 or Huh7 cells was associated with lower resistance to gamma radiation in a clonogenic survival assay which allows for the survival and proliferation of non-stem cancer cells as well as CSCs (Figure 4). This finding is in keeping with previous studies demonstrating that the radioresistance of bulk tumors cells appears to be related to the CSC component of the tumor population [20], [45], [46]. To examine this further, we knocked down Sox2 or Oct4 expression in HepG2 or Huh7 cells using asymmetric-RNA technology and examined their ability to grow as sphere cultures. We found that knockdown of Sox2 or Oct4 expression was associated with a significant decrease in sphere formation following gamma radiation treatment (Figure 5). Since this experiment was performed under stem cell culture conditions, which are selective for CSC enrichment and survival, our results should only reflect the effect of Sox2 and Oct3/4 knockdown on CSCs. Interestingly, Sox2 and Oct3/4 downregulation significantly reduced the sphere forming ability in non-irradiated HepG2 and Huh7 cells, indicating that these factors may also be required for maintenance of existing CSCs. These findings suggest that knockdown of Sox2 and Oct3/4 may be a potential approach for sensitizing hepatocellular carcinomas to radiotherapy since blockade these factors can prevent the self-renewal of non-CSCs that have acquired stemness properties, as well as existing CSCs.

Long-term, non-targeted effects, of ionizing radiation such as genomic instability, adaptive responses and the bystander effect are considered to have a major role in radiation-induced carcinogenesis [66], [67], [68]. Exposure of cells to radiation, especially low doses, can mediate genomic instability and adaptive responses that have the potential to induce gene expression, chromosomal rearrangement, post-translational modifications and epigenetic changes that initiate carcinogenesis. These changes can also be induced in other cells that have not been subjected to initial radiation-damage (by the bystander effect) leading to a more amplified phenotype. Moreover, they are heritable, non-clonal, and rely on epigenetic modifications such as dysregulation of DNA methylation [69], [70], [71]. Our finding that gamma radiation can induce spherogenesis in non-stem cancer cells, and that this process requires the expression of Sox2 and Oct3/4, are consistent with the activation of a “stemness program” mediated by non-targeted epigenetic effects in irradiated cells where the reprogramming of gene expression is associated significantly increased radio-resistance [72].

For the past century, radiation therapy has been used extensively as a curative or adjuvant cancer treatment, and low dose radiation as a palliative measure for managing patients with advanced cancer. However, most human malignancies, including hepatoma, are refractory to this important therapeutic modality. In this study, we show that radiation can induce stem cell-like properties such as sphere formation and stemness gene expression in non-CSCs, demonstrating that non-stem cancer cells can acquire a more stem cell-like state with enhanced ability to self-renew, suggesting a novel mechanism for the radioresistance commonly observed in human malignancies.

## Methods

### Cell Culture and Drug Treatment

HepG2 hepatocellular carcinoma cells were purchased from American Type Culture Collection (HB-8065). Huh7 hepatocellular carcinoma cells were kindly provided by Dr. Raymond Chung [73], [74]. HepG2 and Huh7 human hepatocellular carcinoma cells were cultured in Dulbecco’s modified Eagle’s medium (DMEM; Sigma-Aldrich) supplemented with 10% heat-inactivated fetal bovine serum (FBS; Sigma-Aldrich), 2 mM glutamine, 50 IU/ml penicillin and 50 µg/ml streptomycin in a humidified atmosphere containing 5% CO_2_ at 37°C. To assess sphere formation (spherogenesis) HepG2 and Huh7 cells were cultured in stem cell media (SCM) comprised of DMEM-F12 media, 1× B27 supplement, 200 ng/ml EGF, 10 ng/ml basic FGF, 0.4% BSA, 4 µg/ml insulin, 50 IU/ml penicillin and 50 µg/ml streptomycin in a humidified atmosphere containing 5% CO_2_ at 37°C.

### Gamma Radiation Treatment

Unsorted HepG2 and Huh7 cells and non-SP sorted population suspended in SCM were aliquoted into 1.5 ml tubes at a concentration of 1×10^6^ cells/ml. The tubes were placed on ice and irradiated with 0 Gy, 2 Gy, 4 Gy, 6 Gy, 8 Gy or 10 Gy of gamma radiation using a ^137^Cs irradiator (CIS Diagnostic). The treated cells were then seeded in SCM for spherogenesis assay (at 0.5×10^3^/well), or in complete DMEM for MTT viability assay (at 1×10^3^ cells/well) and colony formation assay (at 3×10^3^ cells/well).

### Hoechst 33342 Staining and Side Population Flow Cytometry

Side Population flow cytometry was performed according to the method of Goodell et al. [75] with modifications to improve the staining for hepatic cell lines. Briefly, HepG2 or Huh7 cultures were trypsinized, and the detached cells were collected by centrifugation at 500 r.p.m. for 5 minutes. The pelleted cells were resuspended at a concentration of 10^6^ cells/ml in pre-warmed DMEM, supplemented with 2% FBS and 10 mM HEPES, containing 5 µg/ml Hoechst 33342 (Sigma-Aldrich) with or without 50 µM verapamil (Sigma-Aldrich). The cells were then incubated at 37°C in a water bath for 90 minutes with gentle mixing every 15 minutes. At the end of the incubation period, the cells were centrifuged at 500 r.p.m. for 5 minutes at 4°C, and resuspended at a final concentration of 2×10^7^ cells/ml in ice-cold Hank’s Buffered Salt Solution supplemented with 0.2% FBS, 10 mM HEPES, 40 µm mesh-filtered and stained with 2 µg/ml propidium iodide. The samples were kept on ice until they were separated using a MoFlo high-speed FACS machine (DakoCytomation) into fractions containing Side Population (SP) cells and non-Side Population (non-SP) cells [6], [32], [33], [34]. Hoechst 33342 was excited using a UV laser at 350 nm and its fluorescence was detected using a 450 nm Hoechst blue filter and a 670 nm Hoechst red filter. Propidium iodide fluorescence was measured using a 650 nm filter. Non-SP and unsorted cells were then transferred into SCM for gamma radiation treatment and analysis of sphere formation. For PI-sorting of bulk cells, HepG2 and Huh7 cells were resuspended at a concentration of 10^6^ cells/ml in pre-warmed DMEM, supplemented with 2% FBS and 10 mM HEPES, then processed as described above. Cells were sorted based only on PI gating and then collected in tubes with SCM for gamma radiation treatment and analysis of sphere formation.

### In vitro Spherogenesis Assay

Non-irradiated and gamma-irradiated unsorted HepG2 and Huh7 cells and non-SP sorted populations were washed three times with SCM to remove all traces of FBS. HepG2 and Huh7 cell suspensions (100 µl) were then plated onto ultra-low attachment 96 well plates (Fisher Scientific) at density of 5 or 10 cells/µl (i.e. 0.5×10^3^ cells/well or 1×10^3^ cells/well respectively) in SCM. Sphere growth was monitored for 5–14 days, and the number of spheres was counted on day 7 and day 14. To keep volume of media in the well constant, ∼25 µl of SCM was added every 4–5 days. At the end of the experiment (day 14) 10 µl of trypan blue dye solution (Sigma-Aldrich) was added to each well to detect dead cells. An average of 6 wells were seeded for each radiation dose.

### Quantitative Real-time PCR

Total RNA was prepared from HepG2 and Huh7 cells 0, 3, 6 and 24 hours after gamma radiation treatment using TRIzol reagent (Invitrogen) according to the manufacturer’s instructions. RNA (5 µg) was then treated with DNAse and reverse transcribed into cDNA using a High Capacity cDNA-Reverse Transcription Kit (Applied Biosystems). TaqMan quantitative real-time PCR for Sox2 and Oct3/4 mRNA was then performed using an ABI 7700 Sequence Detector System (Applied Biosystems) and normalized to GAPDH levels in each sample. TaqMan primers/probes for Sox2, Oct3/4 and GAPDH were purchased commercially (Applied Biosystems). Relative changes in the amount of mRNA were calculated based on the ΔΔCT method.

### Western Blotting

HepG2 and Huh7 cell samples for Western blot analysis of Sox2 and Oct4 protein levels were collected 6 and 24 hours after gamma radiation treatment. Briefly, the cells were lysed in a buffer solution of 2 mM HEPES (pH 6.5) and 2.0% SDS by 3 cycles of boiling for 5 minutes flowed by incubation on ice for 2 minutes. Proteins were then precipitated at −20°C using 60% acetone for at least 2 hours, and centrifuged at 12000 r.p.m. for 20 minutes at 4°C. The protein pellets were the air-dried and resuspended in 80 to 150 µl of lysis buffer, depending on cell pellet size, left at 50°C for 30 min. and then the protein concentration was quantified by using a Micro BCA Protein Assay (Fisher Scientific). Samples containing 8 µg total protein were then separated by 10% SDS-PAGE and transferred to nitrocellulose membranes. The membranes were blocked at room temperature for 1 hour by incubation in TBS containing 0.1% Tween (TBST) containing 5% (w/v) low fat milk. After blocking, the membranes were washed in twice TBST, and then incubated with a rabbit polyclonal human Oct4 antibody (1∶1500; Abcam), a rabbit polyclonal human Sox2 antibody (1∶800; Santa Cruz Biotechnology) or a rabbit polyclonal human α-tubulin antibody (1∶2000; Cell Signaling Technology) in blocking buffer for 1 hour. After washing three times in TBST, the membranes were incubated with an HRP-conjugated anti-rabbit IgG antibody (1∶3000; BioRad) in blocking buffer for 1 hour. After washing three times in TBST, primary antibody binding was visualized by enhanced chemiluminescence and x-ray film. Protein band density was quantified using Image J software.

### Stemness Gene Knockdown

Two aiRNAs [38] targeting Sox2 or Oct4, and one aiRNA targeting GFP were generated using the following sequences:

SOX2 (1): SS, 5′-AAGAGGAGAGUAAGA; AS, 5′-AAUUCUUACUCUCCUCUUUUG

SOX2(2): SS, 5′-AAGAAAACUUUUAUG; AS, 5′-AAUCAUAAAAGUUUUCUUGUC

OCT4(1): SS, 5′-UGAUGCUCUUGAUUU; AS, 5′-AAAAAAUCAAGAGCAUCAUUG

OCT4(2): SS, 5′-GCAUUCAAACUGAGG; AS, 5′-AAACCUCAGUUUGAAUGCAUG

GFP: SS 5′-UAUGUACAGGAACGC; AS 5′-AAUGCGUUCCUGUACAUAACC

aiRNAs (100 nM) were transfected into HepG2 and Huh7 cells using Dharmafect Reagent 4 (Dharmacon, Lafayette, Colorado). After 24 hours, samples were harvested, washed twice in SCM and irradiated with either 0, 2 or 4 Gy. Cell suspensions (100 µl) were then plated into ultra-low attachment 96 well plates (Fisher Scientific) at 0.5×10^3^ cells/well in triplicate, and spheres were counted on Day 7, after addition of 10 µl of trypan blue dye solution. Western Blot assays were performed 48 h after aiRNA transfection to determine knock down efficiency as described above.

### Radiation Survival

HepG2 and Huh7 cell viability after irradiation was determined by MTT (3-(4,5-Dimethylthiazol-2-yl)-2,5-diphenyltetrazolium bromide) assay. LD50 was estimated by non-linear regression first order polynomial equation generated with GraphPad Prism version 4.00, GraphPad Software, San Diego, California, USA.

### MTT Assay

Briefly, HepG2 or Huh7 cells, irradiated by 0 Gy, 2 Gy, 4 Gy, 6 Gy, 8 Gy and 10 Gy, were seeded in quadruplicate onto 96 well plates in complete DMEM at a concentration of 1×10^3^ cells/well and then incubated at 37°C, 5% CO_2_ for 6 days. 10 µl of 5 mg/ml MTT solution (Sigma-Aldrich) were added to each well, in a 1∶10 dilution to media, followed by 5 minutes plate mixing. Plates were then placed in the dark at 37°C for 4 hours. After incubation, media with MTT was discarded from plates and they were dried on a paper towel for few minutes. Formazan crystals formed at bottom of the wells were solubilized in 100 µl DMSO and the plates were mixed for 5 minutes before being scanned in a multiwell spectrophotometer (VersaMax microplate reader, Molecular Devices, Sunnyvale, California) at wavelength of 560 nm. Background optical density was read at 670 nm and subtracted from formazan O.D. All sets of experiments were performed in triplicate for each cell line.

### Colony Formation Assay

For radioresistance evaluation, HepG2 and Huh7 cells were transfected with aiRNA (100 nM) targeting GFP, Sox2 or Oct4, as described above. Cells were collected 24 hours after aiRNA treatment and each sample was divided into 6 aliquots for treatment with 0 Gy, 2 Gy, 4 Gy, 6 Gy, 8 Gy and 10 Gy of gamma irradiation. For each dose, identical numbers of cells were seeded in triplicate onto 6-well plates in complete DMEM. After 7 days, media was removed from wells, colony were washed with PBS twice, before being stained with Hema 3 System (Fisher Healthcare). Wells were then washed with distilled water, the plates were scanned, and images of each well were analyzed using DotCount software v1.1 (Dr. Martin Reuter, http://reuter.mit.edu/software/dotcount/) and number of colonies consisting of at least 50 cells was recorded. A total of 4 independent experiments were performed. LD50 was estimated by non-linear regression first order polynomial equation generated with GraphPad Prism version 4.00, GraphPad Software, San Diego, California, USA.

### Statistical Analysis

All results are presented as mean±SEM. Statistical analysis was performed using unpaired t-test (Figure 1, panels a and b; Figure 4, panels c and d) or one-way ANOVA followed by *post hoc* Dunnett’s test (Figure 2, panels b and c). In Figure 3, panels a to d, and in Figure 5, panels a and b, statistical significance was assessed by one-way ANOVA followed by *post hoc* Dunnett’s test for intergroup comparisons. *P*<0.05 was considered significant. All statistical analyses were performed using GraphPad Prism (version 4.00) software.

## Supporting Information

Figure S1 **HepG2 and Huh7 cell radio-sensitivity increases with gamma radiation dose.** HepG2 cells and Huh7 cells were exposed to increasing doses of gamma radiation and then plated onto 96-well plates for viability evaluation by MTT assay (**A and B**). MTT assay was performed after 6 days of culture and an LD_50_ = 4.33 or LD_50_ = 4.48 Gy were observed for HepG2 and Huh7 cells, respectively. The viable fraction of cells at each radiation dose, expressed as natural log is plotted on the graphs. Lines were fitted using a first-order polynomial regression. Results are presented as the mean±SEM of three independent experiments where each radiation treatment group was seeded in triplicate.
(TIF)
