# Correction: Ionizing Radiation Induces Stemness in Cancer Cells

**DOI:** 10.1371/annotation/15587a16-d9c2-446b-9601-924c2db5c5d9

**Published:** 2013-03-28

**Authors:** Laura Ghisolfi, Andrew C. Keates, Xingwang Hu, Dong-ki Lee, Chiang J. Li

There were errors in both the Funding and Competing interests.

The correct versions of both are:

Funding: This study was supported by the BIDMC Department of Medicine Foundation and Global Research Laboratory Award (Ministry of Education Science and Technology, Korea). The funders had no role in study design, data collection and analysis, decision to publish, or preparation of the manuscript.

Competing interests: The authors have read the journal's policy and have the following conflicts: CJL has a management position at Boston Biomedical, Inc, a company that develops cancer stemness inhibitors. This does not alter our adherence to all the PLOS ONE policies on sharing data and materials. 

