# Supplementary figures and images for: Ionizing Radiation Induces Stemness in Cancer Cells
Source: PLoS One. 2012 Aug 21;7(8):e43628. doi: 10.1371/journal.pone.0043628 (PMC3424153; doi:10.1371/journal.pone.0043628)

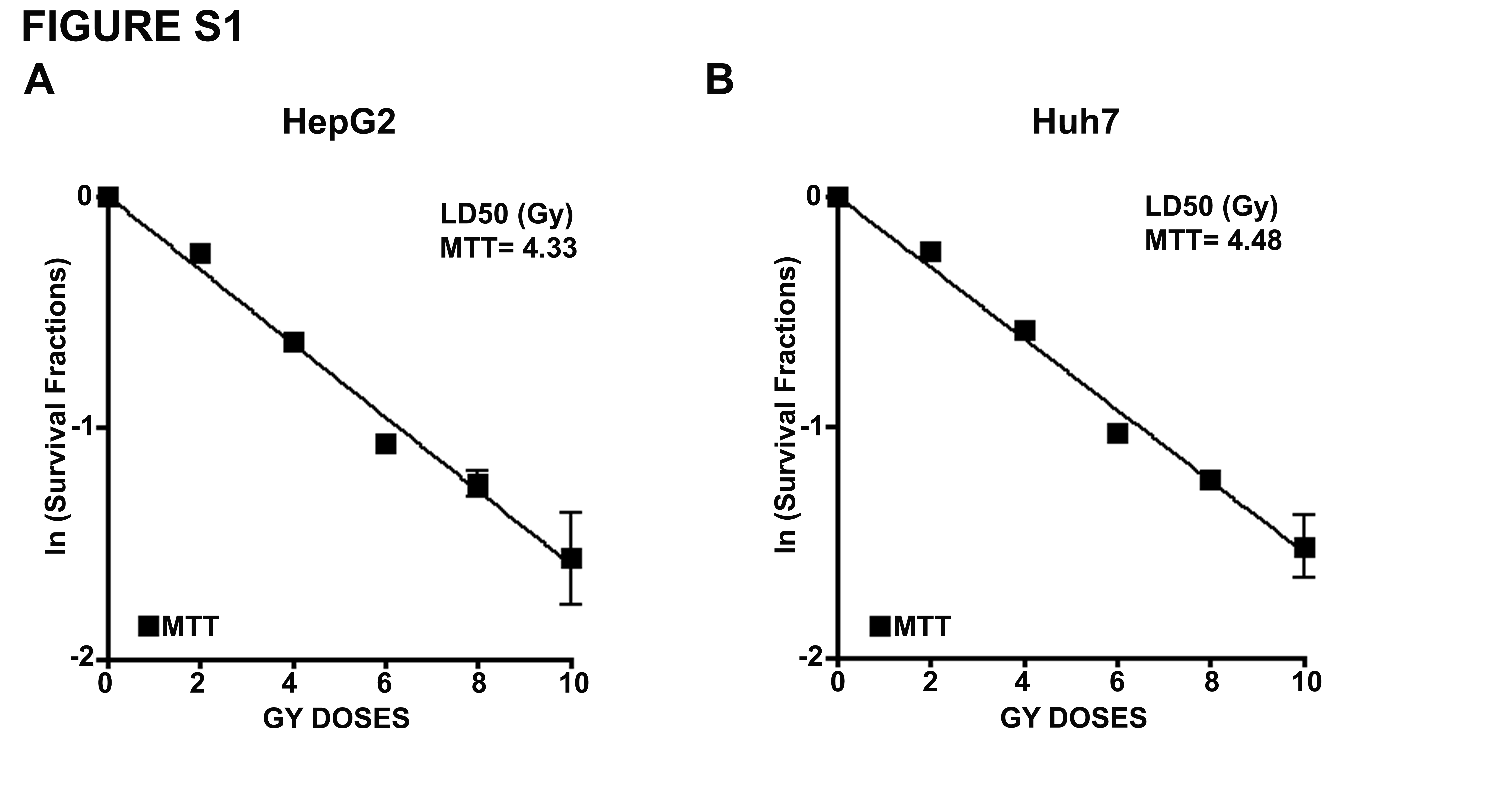

Supplement: Figure S1 — HepG2 and Huh7 cell radio-sensitivity increases with gamma radiation dose. HepG2 cells and Huh7 cells were exposed to increasing doses of gamma radiation and then plated onto 96-well plates for viability evaluation by MTT assay (A and B). MTT assay was performed after 6 days of culture and an LD50 = 4.33 or LD50 = 4.48 Gy were observed for HepG2 and Huh7 cells, respectively. The viable fraction of cells at each radiation dose, expressed as natural log is plotted on the graphs. Lines were fitted using a first-order polynomial regression. Results are presented as the mean±SEM of three independent experiments where each radiation treatment group was seeded in triplicate. (TIF) [file pone.0043628.s001.tif]
